# Statistical Tables of Recoveries

**Published:** 1913-10-11

**Authors:** 


### STATISTICAL TABLES OF RECOVERIES.

### The Debated Value of Recovery Rates.

According to the Annual Report for the year 1912 of the Commissioners in Lunacy, which has just been published, the total number of certified patients admitted to institutions for the insane during the year was: males, 10,716; females, 11,716; total 22,432. The total number of patients discharged as recovered during the year was: males, 3,116; females, 4,229; total 7,345. From these figures are obtained the percentages of recoveries to admissions, which are for males 29.08, for females 36.10, for both sexes 32.74. The percentages varied, of course, in different institutions, and were higher in the registered hospitals than in the county and borough asylums. The percentages in the county and borough asylums were for males 29.06, for females 35.34, for both sexes 32.32; whilst in the registered hospitals they were: for males 36.36, for females 46.03, and for both sexes 42.40. The reason for the higher rate in the registered hospitals is that they deal principally with acute cases, and that most possess some advantage for treatment which the others lack.

The publication of statistics invariably produces a crop of sceptical critics who allege that there is nothing to show that the so-called "recoveries" are truly recovered, and that the majority of them are in reality merely temporarily improved—that the verdict "recovered" depends entirely on the judgment of one man, the medical officer of the institution, and he probably is biassed. To answer this criticism it will be best to glance at a few of the points in regard to the discharges of patients, bearing in mind the fact that the medical officer of an institution has no power to discharge a patient but can only recommend patients as fit to those who have such power.

After a certified patient has been admitted to an institution regular notes have to be made in the case books, and reports have to be sent to the Commissioners in Lunacy at stated intervals. The case-book notes are examined by the Commissioners at their regular visits to the institution. Both the notes and the reports are practically sworn statements by the medical officer who signs them. One of the chief objects of the Lunacy Act being to ensure that no person who is not insane is kept in an institution against his will, and it being the duty of the Commissioners in Lunacy to see that the provisions of the Act are enforced, if these notes and reports are, in the opinion of the Commissioners, insufficient to show that the patient is still insane and a fit and proper person to be kept under care, the medical officer is asked for a further report after a short interval. Should this second report not be considered strong enough, steps are taken as to the patient's discharge.

The Commissioners are fully alive to the necessity of keeping the patient under care during the period of convalescence which follows any mental illness and to the dangers that are likely to follow too early a return to ordinary life and its stresses. The difficulty of doing the best for the patient is mitigated in the registered hospitals, which mostly have convalescent homes attached to them. In the case of the county asylums, however, the difficulty is great, and much greater risks have to be taken. No convalescent establishments are attached to them, and ordinary homes will not take in any case in which the trouble has been mental. The friends are rarely in a position to give any help, and the patient has not only to go straight back home, but has to start working at once in order to earn his own maintenance. Leave of absence on trial can be granted in many cases before actual discharge, but this does not alter the patient's circumstances; it merely means that if a relapse occurs before the expiration of the leave the patient can be sent straight back to the asylum. In some cases the patient is discharged to the Poor-Law authorities, refuses to go to the workhouse, and turns himself adrift upon the world to struggle for existence. The wonder is not that breakdowns occur but that they are not more frequent.

In dealing with private patients, it is a frequent occurrence for the friends to insist on discharge before the medical officer considers the case is well or sufficiently stable to make the chance of a relapse unlikely. When the petitioner acts against expert advice and requests the discharge, if the patient is not at the time physically dangerous to himself and others, the medical officer has no power to refuse. In these cases he is forced to safeguard himself against responsibility and to discharge the patient "Relieved" and not "Recovered." The number of private patients discharged relieved in these circumstances during the year is large.

Should patients who have been under certificates be discharged as "Recovered" unless there are proper surroundings for them to go to which will give them a good chance of keeping well? When the medical officer can find nothing in the conversation or behaviour of the patient which can be taken as proof of mental abnormality, and the patient is anxious to be discharged, there is no power under the Lunacy Act to keep him. How, then, is he to be discharged? Discharged in these cases is forced, and the medical officer has to state at the time of discharge whether it is as "Recovered" or "Relieved." If it is not to be as "Recovered," what is to be the criterion of recovery? Nothing indicating sanity can be found. If a relapse or another similar illness at a later date is to be taken as proof that recovery had not taken place, when can a patient be said to have recovered from any illness, mental or physical? To discharge such patients "Recovered" is a perfectly justifiable procedure.

It should be fully realised, then, that these statistics are based on cases discharged "Recovered" after due consideration, and that the medical officers, though they are handicapped by the Lunacy Act and by financial pressure, fully realise their responsibility in recommending patients to be discharged as "Recovered," and in no case claim a recovery until they have done all in their power to prove it to be one. G.

